# Prostate cancer survivors’ preferences on the delivery of diet and lifestyle advice: a pilot best-worst discrete choice experiment

**DOI:** 10.1186/s40814-019-0549-8

**Published:** 2020-01-06

**Authors:** Luke A. Robles, Stuart J. Wright, Lucy Hackshaw-McGeagh, Ellie Shingler, Constance Shiridzinomwa, J. Athene Lane, Richard M. Martin, Sorrel Burden

**Affiliations:** 1 National Institute for Health Research Bristol Biomedical Research Centre, University Hospitals Bristol NHS Foundation Trust and University of Bristol, Level 3, University Hospitals Bristol Education Centre, Upper Maudlin Street, Bristol, BS2 8AE UK; 2 Manchester Centre for Health Economics, The University of Manchester, Manchester, M13 9PL UK; 3 Manchester Academic Health Sciences Centre (MAHSC), Manchester, M13 9NT UK; 4 North Bristol NHS Trust, Southmead Hospital Bristol, Southmead Road, Westbury-on-Trym, Bristol, BS10 5NB UK; 5 Bristol Randomised Trials Collaboration, Bristol Medical School, Population Health Sciences, University of Bristol, Canynge Hall, 39 Whatley Road, Bristol, BS8 2PS UK; 6 Integrative Cancer Epidemiology Programme, Bristol Medical School, Population Health Sciences, University of Bristol, Oakfield House, Oakfield Grove, Bristol, BS8 2BN UK; 7 School of Health Sciences, Jean McFarlane Building, The University of Manchester, Manchester, M13 9PL UK; 8 Salford Royal NHS Foundation Trust, Salford, M6 8HD UK

**Keywords:** Prostate cancer, Survivorship, Dietary, Lifestyle advice, Discrete choice experiments, Conjoint analysis

## Abstract

**Background:**

Lifestyle factors, including diet and physical activity, are associated with prostate cancer progression and mortality. However, it is unclear how men would like lifestyle information to be delivered following primary treatment. This study aimed to identify men’s preferences for receiving lifestyle information.

**Methods:**

We conducted a cross-sectional pilot best-worst discrete choice experiment which was nested within a feasibility randomised controlled trial. Our aim was to explore men’s preferences of receiving diet and physical activity advice following surgery for localised prostate cancer. Thirty-eight men with a mean age of 65 years completed best-worst scenarios based on four attributes: (1) how information is provided; (2) where information is provided; (3) who provides information; and (4) the indirect cost of receiving information. Data was analysed using conditional logistic regression. Men’s willingness to pay (WTP) for aspects of the service was calculated using an out-of-pocket cost attribute.

**Results:**

The combined best-worst analysis suggested that men preferred information through one-to-one discussion *β* = 1.07, CI = 0.88 to 1.26) and not by email (*β* = − 1.02, CI = − 1.23 to − 0.80). They preferred information provided by specialist nurses followed by dietitians (*β* = 0.76, CI = 0.63 to 0.90 and − 0.16, CI = − 0.27 to − 0.05 respectively) then general nurses (*β* = − 0.60, CI = − 0.73 to − 0.48). Three groups were identified based on their preferences. The largest group preferred information through individual face-to-face or group discussions (*β* = 1.35, CI = 1.05 to 1.63 and 0.70, CI = 0.38 to 1.03 respectively). The second group wanted information via one-to-one discussions or telephone calls (*β* = 1.89, CI = 1.41 to 2.37 and 1.03, CI = 0.58 to 1.48 respectively), and did not want information at community centres (*β* = − 0.50, CI = − 0.88 to − 0.13). The final group preferred individual face-to-face discussions (*β* = 0.45, CI = 0.03 to 0.88) but had a lower WTP value (£17).

**Conclusions:**

Men mostly valued personalised methods of receiving diet and physical activity information over impersonal methods. The out-of-pocket value of receiving lifestyle information was important to some men. These findings could help inform future interventions using tailored dietary and physical activity advice given to men by clinicians following treatment for prostate cancer, such as mode of delivery, context, and person delivering the intervention. Future studies should consider using discrete choice experiments to examine information delivery to cancer survivor populations.

## Background

Prostate cancer is the second most common cancer in men worldwide, accounting for 15% of all male-related cancer diagnoses [1]. It is the fifth most common cause of cancer death worldwide in men, with more than 300,000 deaths per year [2]. In the UK, over 46,000 men are diagnosed with the disease, and there are more than 11,000 deaths annually [3].

Evidence from longitudinal studies suggests that lifestyle factors, such as being overweight or obese, having an unhealthy diet, and little physical exercise can increase a man’s risk of prostate cancer progression. Weight gain over 8-years prior to diagnosis was associated with an increased risk of advanced prostate cancer in men with a body mass index (BMI) ≥ 25kg/m^2^ at age 21 who never smoked [4]. A prospective study including 817 men diagnosed with prostate cancer at a 15 year follow-up observed a positive association between sugar-sweetened beverages and symptomatic prostate cancer [5]. Over 51,500 men who performed ≥ 3 h of vigorous exercise per week had a 61% lower risk of prostate cancer mortality compared to men who performed < 1 h per week over a 10-year follow-up period [6].

The World Cancer Research Fund recommends making changes to nutrition and physical activity behaviours to prevent cancer [7]. These behaviour changes can include increasing fruit and vegetable intake [8], performing 30 minutes of daily moderate to vigorous physical activity [9], and consuming certain nutritional supplements [10]. However, evidence has shown that the majority of cancer survivors do not meet these recommendations [11]. The findings from qualitative work [12] indicated several barriers to the provision of lifestyle advice given by healthcare professionals and adopted by prostate cancer survivors. These barriers included men’s difficulty in understanding written advice or processing a large volume of information, not receiving verbal information from a trusted and credible source (including a nurse or consultant), and men believing lifestyle changes were unnecessary as they were undergoing treatment. Thus, a better understanding of men’s preferences on how lifestyle advice is provided following treatment would be beneficial to both research and clinical practice.

Discrete Choice Experiments (DCE) are being increasingly used in healthcare to elicit participants’ preferences for health care services [13–15]. DCEs are a series of hypothetical scenarios that contain a number of variables or ‘attributes’. Each of these attributes has a number of variations known as ‘levels’. Participants are shown questions containing a number of scenarios, which are created by combining these attributes and levels, and are traditionally asked to choose their most preferred scenario. This produces a binary outcome, whether a particular scenario was chosen or not, which means that logistic regression or probit models can be used to determine which attributes and levels were most likely to predict the chosen scenario [16, 17]. An out-of-pocket cost attribute can be included to represent the inconvenience that participants would experience in accessing the service. For example, patients may have to forgo work or leisure activities in order to attend the health service and this has a cost to them. The inclusion of such attributes allows the calculation of the willingness to pay for attributes and levels. These figures represent the maximum inconvenience (in terms of cost) that participants would be willing to go through to receive beneficial aspects of the service and, therefore, may provide a more intuitive method for valuing the levels of the service than the coefficients of the logistic regression model.

DCEs have evolved to include best-worst (BW) configurations in which participants choose what they think is the best and worst scenarios from each question. Best-Worst Discrete Choice Experiments (BWDCE) have several advantages, which include doubling the amount of data produced within each scenario, capturing more information about preferences at the lower end of the utility scale, and having increased statistical power [18, 19]. The identification of men’s preferences for lifestyle advice is an important consideration for the development of information-based interventions aimed to help men adopt lifestyle changes.

### Aims and objectives

The primary aim of this pilot study was to identify men’s preferences for receiving diet and physical activity lifestyle advice following radical prostatectomy (surgery) for localised prostate cancer. The secondary objectives of this study were to:

- evaluate how difficult men found making their choices;
- identify which attributes they used to make their choices;
- identify which attributes were the most important in their decisions.

## Methods

### Trial design

In this cross-sectional pilot study, participants completed a self-report questionnaire approximately 6 months after radical prostatectomy for localised prostate cancer. This study was nested within a feasibility randomised controlled trial [20], which randomised men to a dietary and physical activity intervention six weeks following surgery for localised prostate cancer.

### Participants and recruitment

Participants were men aged 18 or over, who were diagnosed with localised (low risk) prostate cancer (i.e. cancer that has not spread outside the prostate gland) within the last 12 months, and who had undergone radical prostatectomy. A total of 70 men were approached (51 in person and 19 via post) between February and December 2016. For the majority of men, this was at their six-month follow-up research clinic appointments by the research nurse. Men, who agreed to participate in the BWDCE, provided written informed consent and completed the BWDCE questionnaire. Men completed the questionnaire either during their research clinic appointments or were provided with a freepost envelope to return it to the research team. Recruitment was supplemented by postal questionnaires sent to men who had already completed their 6-month RCT follow-up and who had indicated at consent into the feasibility RCT that they were happy to be approached regarding further research. These men received an information sheet, consent form, and questionnaire to complete and return in a freepost envelope.

### Best-worst discrete choice experiment questionnaire

The BWDCE questionnaire presented each participant with a set of the same 12 hypothetical scenarios. Each scenario consisted of four situations that included four attributes: (1) how information is provided; (2) where information is provided; (3) who provides information; and (4) the indirect cost of receiving information for patients. Table 1 gives an example of a hypothetical scenario. While patients would not be expected to pay for the information they receive, the inclusion of an out-of-pocket cost which represents the inconvenience of receiving information allows the calculation of willingness to pay (WTP) values. This is achieved by calculating the amount of additional cost that men are willing to incur before a service featuring a specific attribute or level before the cost outweighs the benefit they would receive from the service. These monetary values show how valuable each attribute is to the population. The appropriate level of out-of-pocket costs to force participants to trade was determined in the best-worst DCE reported by Wright et al. [21], on which this study was based. In the initial pilot study for that DCE, participants did not use the cost in their decision making, indicating the values were not enough to outweigh the benefits of the health service. Following the doubling of the values for the main study, cost became a significant factor in participants’ choices.

**Table 1:** An example question with different attributes and levels

	Situation 1	Situation 2	Situation 3	Situation 4
How you receive information	Individual face to face discussion	Email	Telephone	Group discussion
Where you receive information	Community centre	Home	Home	Hospital
Who gives you the information	General nurse	Prostate cancer nurse	Dietitian	Dietitian
Indirect cost to you of receiving information	£30	£30	£100	£10
Which do you believe is the best situation (please tick one)	☐	☐	☐	☐
Which do you believe is the worst situation (please tick one)	☐	☐	☐	☐

Each attribute contained four levels which described how or when information is given and by whom. These levels were, then, combined to create a set of 12 questions using a d-efficient design created in NGene software [22] to improve the statistical efficiency of the collected data by minimising the generalised variance of the parameter estimates. This means that the error in measuring preferences introduced by the questionnaire is minimised, thereby improving the coefficient estimates. This also ensures that the coefficients of all attributes can be estimated unlike in random designs where some levels may accidentally not be included. To ensure the realistic nature of each scenario, certain levels of attributes were prevented from appearing together (i.e. telephone calls and emails could only be received in the privacy of their own home). Participants were asked to choose one situation that they most preferred and one situation they least preferred within each scenario. To help with further evaluation of the questionnaire, participants were asked three multiple-choice questions about how they found completing the questionnaire, once they had completed all of the scenarios. These questions were asked: (1) how difficult they found making choices (very easy, easy, neither easy or hard, hard, very hard); (2) which attributes they used in their decision (how the information is given, where the information is given, who gives the information, cost, all of these); and (3) which attribute they found most important in their decision (how the information is given, where the information is given, who gives the information, cost, all of these).

The BWDCE also included questions on participants’ occupation status and household monthly income. Data on other characteristics such as age, marital status, ethnicity, and smoking status were obtained from responses given as part of the feasibility RCT.

The questionnaire was developed and reviewed initially by a group of colorectal survivors (*n* = 32) [21] and, prior to its use in this study, was reviewed by prostate cancer survivors (*n* = 4) from a patient and public involvement (PPI) group to evaluate its acceptability. Minor amendments to the questionnaire were required, which included the wording of the attributes to specify prostate cancer instead of colorectal cancer.

### Sample size

Calculating a minimum sample size for discrete choice experiments poses significant challenges as a number of factors are implicated in determining the size of preference coefficients including: the magnitude of preferences, the number of questions asked, the number of alternatives in each question, and the degree of preference heterogeneity in preferences [23]. In this study a convenience sample was obtained from a larger trial. While this sample was limited in size, previous DCE researchers have suggested that in many cases a sample size of only 20 individuals can serve as a minimum to estimate reliable models [24].

### Statistical analysis

There were three phases to the analysis of the discrete choice data. In the first phase, the factors predicting a profile being chosen as best or worst were analysed separately using conditional logistic regression models which accounted for the grouping of responses by each participant. Analysing the best and worst choices separately serves as a check to determine whether the participants have used the same choice strategy for both answers. There is a lot of information to take on in each DCE question and participants may make choices based on only a few attributes or by other rules such as never choosing profiles containing a certain level. These approaches are known as simplifying heuristics and it has been argued that if preferences are significantly different for best and worst choices then participants may be using such heuristics to make choices rather than fully evaluating all attributes and levels. In such situations it may be inappropriate to combine the best and worst data due to a violation of the underlying assumptions of the choice model.

The second phase of data analysis was to combine the best and worst choice data in a sequential best-worst logistic regression. In this model, the coding of the worst choice is inverted and the data are appended to the best data in a combined dataset. As well as checking for similarities in the best and worst only choices, a second test was also made to check for differences in the consistency of participants in expressing their preferences. Such differences, known as scale heterogeneity or heteroscedasticity, may result in different levels of error variance in the responses to the best and worst questions which can bias the results of combined models. As such a heteroscedastic best-worst logistic regression was used to test for statistically significant differences in scale.

The final phase of data analysis aimed to identify whether there was heterogeneity in participants’ preferences for a healthy lifestyle and dietary intervention. This was achieved using latent class analysis which divides the sample into groups who exhibit similar preferences. To determine how many groups would be appropriate the Bayesian Information Criterion (BIC), a measure of the level of unexplained variation in the data, was observed for increasing numbers of latent classes with the analysis stopped when significant decreases in BIC were no longer achieved.

For each phase of the analysis, willingness to pay (WTP) estimates were also calculated by dividing the coefficient of each level of each attribute by the coefficient of the cost attribute multiplied by minus one. This allows a simpler metric to compare relative preferences for attributes and levels. As the attributes in this BWDCE were effects coded, the WTP values should be interpreted as changes relative to an ‘average’ intervention. For example, positive WTP values mean that participants were willing to pay more for an intervention featuring that level than for a programme of average value.

## Results

### Demographics

Table 2 presents a summary of the characteristics of men included in the analyses. Men were, on average, aged 65 years (SD = 6.04) and all reported themselves as White British or White other (*n* = 38, 100%). The majority were married (*n* = 31, 82%) and employed (*n* = 35, 92%). Over half of men attained higher education (*n* = 22, 58%), reported a monthly income of more than £2000 (*n* = 22, 58%), and that they had never smoked (*n* = 20, 53%).

**Table 2:** Participant characteristics

Characteristics	*N*	%
Mean age, years (SD)	65	(6.04)
Ethnicity		
White British/Other	100	100
Marital status		
Married	31	82
In a relationship	4	11
Separated	1	3
Divorced	2	5
Occupation		
Employed	35	92
Retired	3	8
Education attainment		
Standard education or less	16	42
University degree	14	37
Further education	8	21
Monthly income (£)		
Over 2000	22	58
1001 to 2000	11	29
501 to 1000	1	3
Did not answer	4	11
Smoking status		
Current smoker	1	3
Ex-smoker	15	39
Never smoked	20	53
Missing	2	5

### Analysis of best and worst data

Forty questionnaires were completed and returned to the research team (57% response rate). Data from two men were excluded due to missing or double-answered items to the BWDCE. Data from the remaining 38 questionnaires were used in the analysis.

The best choice data showed that men overall preferred a face-to-face discussion (*β* = 1.04, CI = 0.74 to 1.33, WTP = £124.26) and strongly disliked receiving lifestyle information through emails (*β* = − 0.993, WTP = £-119.25). They had no preference on the location of information delivery. However, they preferred information to be provided by a prostate cancer nurse (*β* = 0.84, CI = 0.65 to 1.04, WTP = £101.35) and not by a general nurse (*β* = − 0.82, WTP = £-97.97).

Similar to the best choice data, the worst choice data showed that men generally favoured face-to-face discussions (*β* = − 1.34, CI = − 1.68 to − 1.01, WTP = £131.28) provided by a prostate cancer nurse (*β* = − 0.57, CI = − 0.76 to − 0.38, WTP = £55.58) over a general nurse (*β* = 0.37, WTP = £-36.07), and disliked receiving information via email (*β* = 1.13, WTP = £-91.47). There was no evidence of a preference on the location of information delivery. In addition, men disliked information delivered through telephone calls (*β* = 0.37, CI = 0.02 to 0.72, WTP = £-36.21) or by a dietician (*β* = 0.20, CI = 0.05 to 0.35, WTP = £-19.51).

There was strong agreement between the best only and worst only data suggesting that it is appropriate to combine the data. However, there was some evidence to suggest that telephone calls may be both disliked and liked (although not statistically significant). This may indicate the presence of potential preference heterogeneity.

### Analysis of combined best-worst data

The results of the sequential best-worst logistic regression (Table 3) showed similar results to those reported in the separated analysis of the best-worst choices. A heteroscedastic best-worst logistic regression model suggested that there was no difference in error variance between the best and worst choices (*β* = 0.04, *p* = 0.73) or between patients who completed a questionnaire in person or at home (*β* = − 0.09, *p* = 0.45). Thus, combining the data was appropriate. Individual face-to-face discussion (*β* = 1.07, CI = 0.88 to 1.26, WTP = £107.73) was still the most important attribute with a dislike for emails (*β* = − 1.02, WTP = £-102.42). Men preferred a prostate cancer nurse (*β* = 0.76, CI = 0.63 to 0.90, WTP = £76.74) to provide the lifestyle information over a dietician (*β* = − 0.16, CI = − 0.27 to − 0.05, WTP = £-15.89) or general nurse (*β* = − 0.60, WTP = £-60.85). There was no evidence of a preference on where the information was given.

**Table 3:** Combined best and worst case data

Attribute/level	Unstandardised beta	LCI	UCI	*p*	WTP (£)
How information is provided					
Telephone	− 0.105	− 0.296	0.086	0.281	− 10.57
Individual Discussion	1.070**	0.884	1.256	<0.001	107.73
Group discussion	0.052	− 0.166	0.271	0.639	5.26
Email	− 1.017**	− 1.234	− 0.800	<0.001	− 102.42
Where information is provided					
Hospital	− 0.012	− 0.205	0.181	0.905	− 1.18
GP	0.073	− 0.098	0.245	0.402	7.38
Community Centre	− 0.094	− 0.256	0.069	0.260	− 9.43
Own Home	0.032	− 0.221	0.285	0.804	3.22
Who provides information					
Prostate cancer nurse	0.762**	0.627	0.897	<0.001	76.74
Dietitian	− 0.158*	− 0.267	− 0.049	0.004	− 15.89
General nurse	− 0.604**	− 0.727	− 0.482	<0.001	− 60.85
Cost of receiving information	− 0.010**	− 0.012	− 0.008	<0.001	

*LCI* = lower 95% confidence interval; *UCI* = upper 95% confidence interval; *WTP* = willingness to pay; *p ≤ 0.01; **p ≤ 0.001

### Latent preference classes for advice provision

A latent class sequential best-worst logistic regression identified three distinct groups based on their preferences, but not on their demographics (Table 4). The largest group (n = 20, 53%), labelled ‘face to face communicators’, preferred information through individual (*β* = 1.35, CI = 1.06 to 1.64, WTP = £140.08) or group discussions (*β* = 0.70, CI = 0.38 to 1.03, WTP = £73.10) and not via the telephone (*β* = − 0.53, CI = − 0.823 to − 0.239, WTP = £-55.26) or email (*β* = − 1.52, CI = − 1.86 to − 1.18, WTP = £-157.93). The second group (n = 10, 26%), labelled ‘one to one communicators’, wanted to receive information via telephone calls (*β* = 1.03, CI = 0.58 to 1.48, WTP = £134.00) or individual face-to-face discussions (*β* = 1.89, CI = 1.41 to 2.37, WTP = £245.22), but did not want information from group discussion (*β* = − 1.54, CI = − 2.08 to − 1.00, WTP = £-199.94), by email (*β* = − 1.38, CI = − 1.89 to − 0.87, WTP = £-179.27), or in community centres (*β* = − 0.50, CI = − 0.877 to − 0.131, WTP = £-65.49). The final group (n = 8, 21%) did not have strong preferences on where the information was provided but were particularly sensitive to the indirect cost attribute and as such were labelled ‘cost-sensitive communicators’. Members of this group preferred individual face-to-face discussions (*β* = 0.45, CI = 0.03 to 0.88, WTP = £17.24), but were not willing to pay more than the other two groups for it. All groups preferred a prostate cancer nurse, as opposed to a general nurse, to provide the information. In addition, the cost-sensitive group expressed less of a preference for dieticians’ involvement.

**Table 4:** Best and worst cases by group preferences

	Face to face communicators (*n* = 53%)					One to one communicators (*n* = 26%)					Cost-sensitive communicators (*n* = 21%)				
Attribute/levels	Unstandardised beta	LCI	UCI	*p*	WTP (£)	Unstandardised beta	LCI	UCI	*p*	WTP (£)	Unstandardised beta	LCI	UCI	*p*	WTP (£)
How information is provided															
Telephone	− 0.531***	− 0.823	− 0.239	<0.001	− 55.26	1.032***	0.579	1.484	<0.001	134.00	− 0.249	− 0.744	0.246	0.325	− 9.48
Individual discussion	1.346***	1.055	1.636	<0.001	140.08	1.888***	1.408	2.367	<0.001	245.22	0.453*	0.026	0.879	0.038	17.24
Group discussion	0.702***	0.377	1.028	<0.001	73.10	− 1.539***	− 2.075	− 1.003	<0.001	− 199.94	− 0.123	− 0.686	0.441	0.670	− 4.67
Email	− 1.517***	− 1.859	− 1.175	<0.001	− 157.93	− 1.380***	− 1.890	− 0.870	<0.001	− 179.27	− 0.081	− 0.605	0.442	0.761	− 3.10
Where information is provided															
Hospital	0.063	− 0.224	0.350	0.667	6.55	− 0.107	− 0.529	0.316	0.621	− 13.83	− 0.002	− 0.500	0.496	0.993	− 0.08
GP surgery	0.038	− 0.205	0.280	0.759	3.95	0.162	− 0.246	0.569	0.437	20.99	0.311	− 0.150	0.772	0.186	11.86
Community centre	0.056	− 0.181	0.292	0.644	5.79	− 0.504**	− 0.877	− 0.131	0.008	− 65.49	− 0.101	− 0.550	0.348	0.660	− 3.84
Own home	− 0.157	− 0.537	0.224	0.420	− 16.29	0.449	− 0.091	0.989	0.103	58.34	− 0.208	− 0.855	0.439	0.528	− 7.94
Who provides information															
Bowel cancer nurse	0.739***	0.534	0.944	<0.001	76.94	0.479**	0.172	0.785	0.002	62.16	1.872***	1.476	2.268	<0.001	71.33
Dietician	0.058	− 0.109	0.225	0.500	6.03	0.155	− 0.082	0.391	0.200	20.06	− 1.239***	− 1.579	− 0.899	<0.001	− 47.18
General nurse	− 0.797***	− 0.998	− 0.596	<0.001	− 82.97	− 0.633***	− 0.913	− 0.353	<0.001	− 82.22	− 0.634***	− 0.941	− 0.326	<0.001	− 24.14
Cost of receiving information	− 0.010***	− 0.013	− 0.006	<0.001		− 0.008**	− 0.013	− 0.002	0.005		− 0.026***	− 0.033	− 0.019	<0.001	

*LCI* = lower 95% confidence interval; *UCI* = upper 95% confidence interval; *WTP* = willingness to pay; *p ≤ 0.05; **p ≤ 0.01; ***p ≤ 0.001

### Evaluation of the BWDCE questionnaire

Nearly half of the men (*n* = 17, 45%) reported the BWDCE to be ‘quite hard’ to complete whilst a relatively small number of men (*n* = 7, 18%) reported that they found it ‘quite easy’ to complete. Over half of the men (*n* = 21, 55%) considered ‘who gives the information’, followed by ‘how the information was given’ (*n* = 18, 47%), ‘where the information was given’ (*n* = 11, 29%), and ‘the cost’ (*n* = 6, 16%) when making their decision. Half of the men (*n* = 19, 50%) reported ‘who gives the information’ followed by ‘how the information was given’ (*n* = 7, 18%) as the most important attribute in their decision.

## Discussion

This study aimed to identify men’s preferences for receiving diet and physical activity information following radical prostatectomy for prostate cancer. The findings showed that men were not particularly concerned with where the information was provided. However, there was near universal agreement that prostate cancer nurses should provide the information, not general nurses, and this was the attribute selected as most important by the largest number of participants. The findings from the latent class analysis showed that information delivery may need to be stratified for different groups of men (i.e. via face-to-face, group discussion, or telephone call). Furthermore, some men considered the indirect cost of receiving lifestyle information and so minimising the potential financial implications of receiving information may be an important consideration.

With growing popularity of DCEs in recent times, few studies have employed this method to examine preferences of diet and physical activity information delivery in cancer survivors. Nevertheless, evidence from available studies somewhat supports the findings from this study. Wright and colleagues [22] used the same BWDCE questionnaire to quantify the preferences for lifestyle information delivery in 179 men and women adult survivors of colorectal cancer, who had completed treatment. Patients were recruited from hospital follow-up clinic appointments and completed the BWDCE questionnaire. Similar to this study, the results showed that, overall, survivors preferred face-to-face discussions and preferred the information to be delivered by clinical nurse specialists. However, colorectal survivors mostly preferred telephone discussions and information given in hospitals. In addition, sub-group analyses showed three distinct groups of survivors characterised by method of information delivery. There were also differences found between groups on certain demographic and clinical factors (i.e. age, level of disease risk), which were not possible to analyse in this study.

The present findings suggest that preferences of mode of information delivery may differ among different cancer populations. In a qualitative study [25], men diagnosed with prostate cancer discussed being dissatisfied with dietary advice from various sources, in particular from the internet and the media, as they found it conflicting and lacked a sufficient evidence-base. This was considered a barrier to making dietary changes. Men have suggested that dietary information would need to be tailored toward their own personal circumstances and provided by a trusted source, such as a healthcare professional [26]. In addition, Wright and colleagues’ colorectal study [21] included both men and women suggesting their findings may indicate gender differences in information delivery (e.g. women favouring telephone calls more so than men). Thus, evidence suggests that mode of lifestyle information delivery to men with prostate cancer would benefit from a verbal communication component to accommodate the individual needs of men.

A potential limitation of this study is that all the men reported themselves as White, which makes generalisation of the results problematic. This was due to men recruited from a relatively small sample (~ 100) included in a feasibility RCT in which the majority reporting themselves as White British. Thus, the results would only be generalisable to men of this ethnic group. This may explain why demographic information was not found to predict preference group membership, a factor that would also make personalisation of the intervention difficult in clinical practice. A larger more diverse sample would provide the opportunity to examine differences in sample characteristics and allow stronger interpretations of the findings. If the preferences of this larger sample are similar to that of the study population then the coefficients will be estimated with greater accuracy. However, this may introduce a more heterogeneous population that does not increase the statistical significance of the coefficients due to preference heterogeneity. In this case, the use of latent class analysis, such as that reported in this study, would become more reliable and critical in order to provide evidence as to how the intervention could be tailored to best meet patients’ preferences. Nearly half of the participants in this study reported that they found completing the BWDCE quite difficult. It is possible that participants could have been basing their decisions on specific attributes of the scenario rather than the whole scenario. The absence of an opt-out item meant that, in some cases, participants might have been forced to choose a best choice when there was no favoured option or a worst choice with potential implications for the results [27].

There are several implications of the findings of this study. First, they could help inform studies using healthy lifestyle interventions as DCEs are able to capture data on attributes, such as mode of delivery, context, and person delivering the intervention, that need to be considered when planning an intervention. However, further work might need to evaluate the reliability of the BWDCE. A larger study may be necessary to ensure the preferences of the men in this study are representative of those in the wider population of prostate cancer survivors. Further qualitative interviews may be also useful to determine the acceptability of the results in guiding clinical practice. Second, future interventions may need to consider tailoring information delivery according to men’s preferences on how they would like to receive the information. For example, the feasibility RCT within which this data was collected asked men for their preferred method of contact (i.e. telephone, email, and letter) by the research team at the beginning of the trial to provide lifestyle advice throughout the intervention period. This may help to overcome existing barriers associated with information delivery to patients, increase the likelihood of men adopting changes to lifestyle behaviours, and highlight the importance of adopting these changes on long-term health outcomes. Personalised information delivery could help facilitate a more efficient use of healthcare resources. However, future studies would need to conduct RCTs of different methods of providing lifestyle information, as well as perform a health economic evaluation, to examine the potential benefits gained from personalising lifestyle information on reductions in cancer recurrence compared to the additional costs incurred from employing such methods.

To the authors’ knowledge, this is the first DCE to examine the preferred methods of delivering lifestyle information to men following treatment for prostate cancer. Thus, this study has demonstrated the benefits of using such a method within a prostate cancer population.

## Conclusions

The findings from this study suggest that men valued personalised methods of receiving diet and physical activity information over more impersonal methods, such as information sent via email. The out-of-pocket cost value of receiving lifestyle information was important to some men but was not a key attribute in many of the men’s choices. Future studies would benefit from using DCEs to elicit preferences among much larger samples of cancer patients.

## Data Availability

The datasets generated during and/or analysed during the current study are not publicly available. However, investigators who would like access to specific parts of the data should contact the corresponding author.

## Abbreviations

BIC: Bayesian Information Criterion
BWDCE: Best Worst Scaling Discrete Choice Experiment
PrEvENT: Prostate cancer - Evidence of Exercise and Nutrition Trial
WTP: Willingness to pay
